# Maternal Occupational Risk Factors and Preterm Birth: A Systematic Review and Meta-Analysis

**DOI:** 10.3389/phrs.2023.1606085

**Published:** 2023-10-23

**Authors:** Haimanot Abebe Adane, Ross Iles, Jacqueline A. Boyle, Asmare Gelaw, Alex Collie

**Affiliations:** ^1^ School of Public Health and Preventive Medicine, Faculty of Medicine, Nursing and Health Sciences, Monash University, Melbourne, VIC, Australia; ^2^ Eastern Health Clinical School, Faculty of Medicine, Nursing and Health Sciences, Monash University, Melbourne, VIC, Australia

**Keywords:** pregnancy, systematic review, meta-analysis, preterm birth, occupational risks

## Abstract

**Objective:** This systematic review and meta-analysis aimed to summarize the evidence on the relationship between physical occupational risks (high physical workload, long working hours, shift work, whole-body vibrations, prolonged standing, and heavy lifting) and preterm birth.

**Methods:** A systematic review and meta-analysis was conducted across six databases to investigate the relationship between physical occupational risks and preterm birth.

**Result:** A comprehensive analysis of 37 studies with varying sample sizes found moderate evidence of positive associations between high physical workload, long working hours, shift work, whole-body vibration, and preterm birth. Meta-analysis showed a 44% higher risk (OR 1.44, 95% CI 1.25–1.66) for preterm birth with long working hours and a 63% higher risk (OR 1.63, 95% CI 1.03–2.58) with shift work.

**Conclusion:** Pregnant women in physically demanding jobs, those working long hours or on shifts, and those exposed to whole-body vibration have an increased risk of preterm birth. Employers should establish supportive workplaces, policymakers implement protective measures, healthcare providers conduct screenings, and pregnant women must stay informed and mitigate these job-related risks.

**Systematic Review Registration**: [https://www.crd.york.ac.uk/prospero/], Identifier [CRD42022357045].

## Introduction

The World Health Organization (WHO) defines preterm birth as the birth of a baby before 37 weeks of pregnancy [1]. Rates of preterm birth range from 5% to 18% across 184 nations [2]. An estimated 15 million preterm births occur worldwide each year, with 1.1 million infant deaths as a result of preterm birth, making it one of the leading causes of mortality in children under 5 years of age [3]. Preterm birth can cause short- and long-term health problems for children, such as diabetes, high blood pressure, and heart disease later in life [4–6]. Most preterm births are spontaneous, but around 30% are provider-initiated, involving induction or primary cesarean section, termed medically indicated [7].

The global workforce has seen a significant increase in the participation of pregnant women [8]. In the European Union, two-thirds of women of working age or older were employed in 2020 [9]. Over 40% of women in Europe worked in physically demanding jobs, 21% worked rotating shifts, 15% worked more than 40 h per week, and 14% worked night shifts [9]. In many lower and middle-income countries, the employment rate of women is also high, at 32.17% [10]. However, the vast majority of women who work in the paid economy are in the informal economy [10]. The increasing number of reproductive-age women in paid employment raises concerns about the impact on pregnancy outcomes [11]. Previous studies have shown that pregnant working women are at increased risk of poor maternal and newborn health, including preterm birth [12–14].

Preterm birth is most commonly caused by factors such as multiple pregnancies, infections, and chronic health conditions [15]. However, there is growing evidence that occupational factors, such as physically demanding work, whole-body vibration, long hours, and shift work, may also increase the risk of preterm birth [16–18]. For example, a systematic review of studies found that women who worked long hours were more likely to have a preterm birth [18]. Another review found that pregnant women who worked long hours while standing, lifting heavy objects, or working shifts or nights were also at increased risk [16, 19].

While the evidence from these reviews is useful, their authors report conflicting or weak evidence and as such have concluded that it is challenging to provide explicit recommendations for clinical practice or policy [12, 16, 18]. Some limitations of these prior reviews include not reporting on study quality [12, 20], none have examined the impacts of whole-body vibration on preterm birth, and none have sought to differentiate between medically indicated or spontaneous preterm birth [16, 17, 20]. Further, the included evidence in most reviews reflect working conditions of the late 20th century, up to the early 2000’s [16, 21]. In many occupations and nations, working conditions have changed dramatically throughout the early 21st century and thus the nature, prevalence and impacts of occupational physical health risks has also changed [22, 23].

Pregnant women are often exposed to physical occupational risks, such as high physical workload, heavy lifting, long working hours, long-standing hours, and shift work [22]. These risks are common, have a significant impact on reproductive health [23], and are more modifiable than chemical and biological exposures [21]. This systematic review and meta-analysis was conducted to investigate the relationship between physical occupational risks and preterm birth. A better understanding of this relationship has been gained and is helpful for obstetricians, occupational health services, employers, and policymakers in developing strategies to reduce the risk of preterm birth.

## Methods

This systematic review and meta-analysis was reported in accordance with PRISMA guidelines [24]. The study protocol was registered with PROSPERO (CRD42018094400) and published in PLOS One [25].

### Search Strategy

Six electronic databases were searched without geographic restrictions to identify studies examining the effects of exposure to physical occupational risks, such as physically demanding work, long working hours, shift work, whole-body vibration, prolonged standing, and heavy lifting on preterm birth in paid employed pregnant women. A broad range of potential search terms, including Medical Subject Headings (MeSH) terms and keywords (as shown in Supplementary Table S1), were employed for the search. Additionally, the reference lists of the included studies were examined to identify relevant research.

### Eligibility Criteria

This review included original research studies that examined the link between physical occupational risks and preterm birth in pregnant women who were employed during pregnancy. Studies were observational (prospective, retrospective, case-control, cross-sectional) or interventional designs. Studies were excluded if they were reviews, case studies, qualitative studies, editorials, commentaries, conference abstracts, or unpublished manuscripts; published in languages other than English, before the year 2000, and investigated the effect of non-physical occupational risks, such as biological, chemical, or psychosocial hazards.

### Outcome

The primary outcome of interest was preterm birth, defined as babies born alive less than 37 weeks of pregnancy [1]. We also examined different types of preterm birth as secondary outcomes, including extremely preterm birth (<28 weeks), very preterm birth (28-<32 weeks), moderate preterm birth (32-<37 weeks), and spontaneous birth (delivery onset by spontaneous labor or premature rupture of membranes) or medically indicated birth (delivery onset through induction or primary caesarean section) [7].

### Exposure

Six of the most commonly prevalent physical occupational risks were identified as the exposure of interest. These were high physical workload, long working hours, shift work, whole-body vibrations prolonged standing, and heavy lifting. Due to a wide variation in exposure definitions in the literature, we adopted broad definitions to ensure that all articles reporting relevant exposures were captured (See Table 1).

**TABLE 1:** Definition of physical occupational risks (Australia, 2023).

Type of occupational exposure	Definition of exposure
Prolonged standing	Standing more than 3 h per day at work
Heavy lifting	Lifting more than 5 kg at a time or greater than 50 kg per day
High physical workload	A job that requires heavy physical effort or physical exertion, as indicated by at least 1 of the following criteria [1]: Job to the highest physical exertion score category on a standardised scale (such as Job Characteristic Scoring System or dictionary of occupational title physical exertion measures)
	[2] Job combines ≥2 physically demanding tasks (e.g., standing, lifting, and bending)
Long working hours	At least one of the following [1] Working more than 40 h per week
	[2] Working more than 5-days per week
	[3] Working more than a standard 8-h work per day
Shift work	Working hours that rotate or change according to a set schedule
Whole-body vibration	Either of the following [1] Vibrations that are transmitted through the entire body from sitting, standing, or lying on a vibrating surface
	[2] Vibrations exceeding the exposure limit of ≥0.5 m/s^2^

### Study Selection

All articles found from electronic databases and reference chaining were gathered in EndNote. Duplicate articles were removed, and the remaining articles were imported into Covidence. Two independent reviewers screened the titles and abstracts of all articles against eligibility criteria (HAA and AG). Articles on which both reviewers agreed were excluded or progressed to the next stage. Disagreements were resolved by consensus or a third reviewer (RI). The full text of all articles that passed the initial screening was retrieved and assessed for eligibility by two independent reviewers. Again, disagreements were resolved by consensus or a third reviewer.

### Data Extraction

Data were extracted from all included studies by two independent reviewers using a standard data extraction tool. The following information was extracted: study characteristics (study period, study design, country), population characteristics (number of participants), type of exposure, gestational time women engaged in work (exposure timing), method of exposure assessment, outcome (preterm birth and subtype), confounders considered, effect estimates, and main finding.

### Risk of Bias (ROB) Assessment

The risk of bias of the included studies was assessed using tools from the Joanna Briggs Institute (JBI) [26]. These tools assessed the quality of different types of studies for potential sources of bias, such as inappropriate sampling, measurement, outcomes, confounding factors, and statistical analysis. The quality assessment was conducted independently by two reviewers (HAA and AG). In cases where there was a discrepancy, a third reviewer (RI) was consulted to achieve consensus. A study was deemed to have a low risk of bias if more than 70% of responses were marked as “yes,” a moderate risk of bias if between 50% and 69% of responses were marked as “yes,” and a high risk of bias if less than 50% of responses were marked as “yes” [27]. Studies with a high risk of bias were excluded from further synthesis and analysis.

### Evidence Synthesis

We used the GRADE (Grading of Recommendations, Assessment, Development, and Evaluation) method to assess the quality of evidence for each exposure and outcome [28]. The certainty of evidence was rated high, moderate, low, or very low. We started with a high rating for RCTs and a low rating for observational studies. The certainty of evidence from observational studies may be downgraded if two or more of the following five factors are present: risk of bias, indirectness, inconsistency, imprecision, and publication bias. Risk of bias across studies was rated as serious when ≥50% of the eligible studies had high ROB, otherwise it was considered as not serious. Indirectness was rated as serious when ≥50% of the eligible studies had significant differences in the population, exposure or outcomes examined, otherwise it was considered as not serious. Inconsistency was rated as serious when ≥50% of the eligible studies had a large variation in the effect estimate, otherwise it was considered as not serious. Imprecision was rated as serious if ≥ 50% of the eligible studies did not meet optimal information size (OIS) criteria (i.e., if the total number of populations included in the SLR is less than the number of populations generated by a conventional sample size calculation for a single study adequately powered trial), and if OIS was met and the 95% CI overlaps no effect, otherwise it was considered as not serious. Publication bias was rated serious if the eligible studies only included large sample size (≥2000), only reported positive results, and search strategies were believed to be less comprehensive. Otherwise it was considered as not serious. The certainty assessment could also be up-rated if one of three domains were observed (large magnitude of effect, evidence of a dose-response relationship, and counteracting plausible residual bias). The GRADE method was used to develop practical guidance from the evidence [29]. Recommendations were made based on how confident we were in the evidence. High-quality evidence led to strong recommendations, moderate-quality evidence led to practice considerations, and low-quality evidence meant that there was not enough evidence to guide policymakers, clinicians, and patients.

### Meta-Analysis

Meta-analyses were performed using the generic inverse variance method with random effects modelling if there were sufficient studies with a similar definition of exposure and outcomes of interest. We calculated a pooled odds ratio (OR) with a 95% confidence interval (CI) for the primary outcome. Visual inspection of forest plots and I^2^ statistics tests were used to assess heterogeneity between studies. Publication bias was investigated using the Egger’s weighted regression test and the Begg’s test. The meta-analysis was conducted using Stata V17 (Stata/SE, Windows, macOS, Linux).

## Results

### Search Result

**FIGURE 1:**
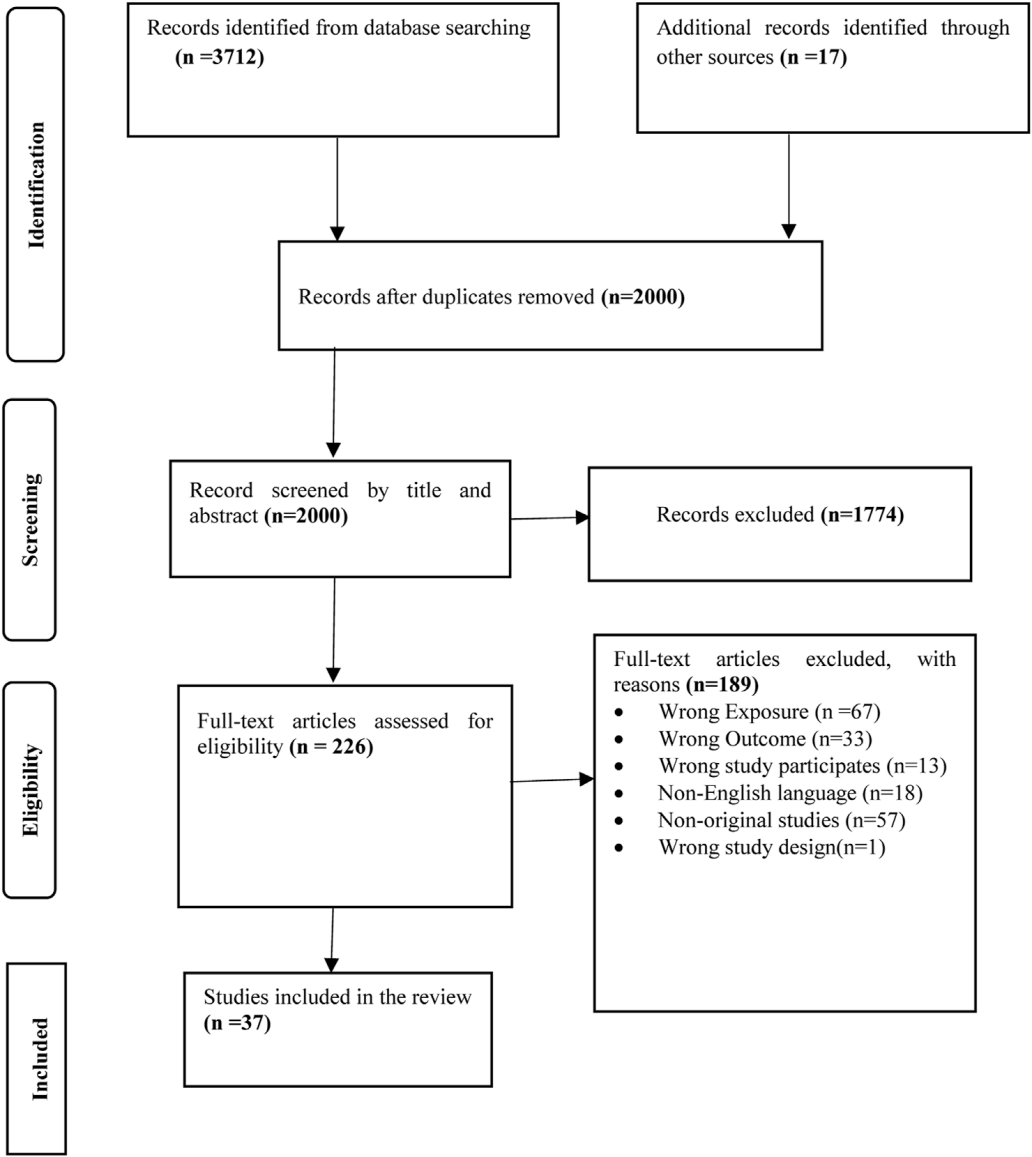
PRISMA flow diagram of searching, screening, and sorting (Australia, 2023).

In the initial search, 3,712 records were identified (See Figure 1). After removing duplicates, screening the title, abstracts and full text, 36 studies were included. One additional study was added from 17 other records identified from the reference lists of included studies. Thus, a total of 37 articles proceeded to data extraction and quality assessment.

### Characteristics of the Included Studies

#### Country of Origin

Table 2 presents the summary of the study characteristics of the 37 included studies. There were 29 studies from high income countries, including 18 studies from Europe [30–47], seven studies from the United States [48–54], two studies from Asia [55, 56] and one each from Australia [57] and Canada [58]. There were fewer (*n* = 8) studies conducted in low-income countries, including four each from Africa [59–62] and Asia [63–66].

**TABLE 2:** Details of included studies from 1 January 2000–September 2022 (Australia, 2023).

Author (Year) Location	Study period	Study design	Sample size	Exposure(s)	Exposure timing	Method of exposure assessment	Outcome(s)	Main findings	Significance
Abeysena et al. (2010) [63] Sri Lanka	2001–2002	Prospective	885	Standing	12 weeks	Interview during pregnancy	Preterm birth (<37 weeks)	Prolonged standing during 1st trimester [COR 1.34 (95% CI 0.71–1.81)], 2nd trimester [COR 0.80 (95% CI (0.47–1.35)], 3rd trimester [COR 0.80 (95% CI 0.46, 1.46)] of pregnancy was not associated with preterm birth	NS
					28 weeks				
					36 weeks				
Agbla et al. (2006) [59] Benin	2000–2002	Case- control	203	Lifting Working hours	Not sated	Interview during postpartum	Preterm birth (<37 weeks)	Heavy lifting [AOR: 5.01 (95% CI 1.38–18.8)], and physical workload [AOR: 6.88 (1.45–32.2] were positively associated with preterm birth	Sig
Arafa et al. (2007) [60] Egypt	2004–2005	Cross-sectional	730	Shift work Standing	Not stated	Interview during postpartum	Preterm birth (<37 weeks)	Shift work (X^2^ = 0.22, *p* = 0.63) and standing posture (X^2^ = 0.02, *p* = 0.99) was not associated with preterm birth	NS
Bell et al. (2008) [48] USA	1979–2000	Prospective	2,508	Physical workload	13 weeks	Job exposure matrix	Preterm birth (<37 weeks)	High physical workload was positively associated with preterm birth [AOR: 1.16 (95% CI 1.03–1.30)]	Sig
Bonzini et al. (2009) [30] United Kingdom	1999–2003	Prospective	1,327	Standing Lifting Working hours Shift work	11 weeks 19 weeks 34 weeks	Interview during pregnancy	Preterm birth (<37 weeks)	Prolonged standing during 1st trimester [AOR 0.92 (95% CI 0.49–1.70)], 2nd trimester [AOR 0.76 (95% CI 0.39–1.49)], 3rd trimester [AOR 0.99 (95% CI 0.39–2.51)] of pregnancy was not associated with preterm birth	NS
								Heavy lifting during 1st trimester [AOR 0.69 (95% CI 0.21–2.26)] and 2nd trimester [AOR 1.10 (95% CI 0.33–3.63)] of pregnancy was not associated with preterm birth	
								Long working hours during 1st trimester [AOR 1.03 (95% CI 0.49–2.15)] and 2nd trimester 1.01(95% CI 0.47–2.17) of pregnancy was not associated with preterm birth	
								Night shift work during 1st [AOR 1.14 (95% CI 0.43–2.93)], and 2nd trimester of pregnancy [AOR 1.07 (0.37–3.05)] was not associated with preterm birth	
Both et al. (2010) [31] UK	1991–1992	Prospective	11,737	Shift works	3rd trimester	Interview during pregnancy	Preterm birth (<37 weeks)	Night shiftwork was negatively associated with preterm birth [AOR 0.67 (95% CI 0.47–0.95)]	Sig^
Burdorf et al. (2011) [32] Netherlands	2002–2006	Prospective	6,302	Standing	Not stated	Interview during pregnancy	Preterm birth (<37 weeks)	Prolonged standing was not associated with preterm birth [AOR 0.86 (95% CI 0.62–1.18)]	NS
Celikkalp et al. (2017) [55] Turkey	2013–2014	Prospective	127	Standing	Not stated	Interview during pregnancy	Preterm birth (<37 weeks)	Prolonged standing [COR = 10.1, *p* = 0.005], long working hours [COR 2.42, *p* = 0.030], and shift work [COR 3.18, *p* = 0.014] were positively associated with preterm birth	Sig
				Working hour					
				Shift work					
Croteau et al. (2007) [58] Canada	1997–1999	Case-control	4,721	Standing	1st trimester	Interview during pregnancy	Preterm birth (<37 weeks)	Prolonged standing [AOR 1.0 (95% CI 0.7–1.7)], and heavy lifting [AOR 0.9 (95% CI 0.6–1.3)], shift work [AOR 1.0 (95% CI 0.9–1.3)] during 1st trimester of pregnancy was not associated with preterm birth	NS
				Lifting					
				Working hour					
				Whole body vibration Shift work				Whole-body vibration [AOR 1.4 (1.1–1.9)], and long working hours [AOR 1.6 (95% CI 1.1–2.4)] during 1st trimester were positively associated with preterm birth	Sig
Davari et al. (2018) [64] Iran	2017	Cross-sectional	429	Shift work	Not stated	Interview during postpartum	Preterm birth (<37 weeks)	Shift work was positively associated with preterm birth [AOR 2.26 (95% CI 1.4–3.5)]	Sig
El-Gilany et al. (2016) [61] Egypt	2014–2015	Cross-sectional	1,340	Lifting	Not stated	Interview during postpartum	Preterm birth (<37 weeks)	Heavy lifting [AOR 2.76 (95% CI 1.98–8.74)], and long working hours [AOR 2.36 (95% CI 1.18–7.78)] were positively associated with preterm birth High physical workload was positively associated with preterm birth [AOR 3.94 (95% CI 1.03–18.19)]	Sig
				Working hours					
				Physical workload					
Escribà-Agüir et al. (2001) [33] Spain	1995–1996	Case- control	576	Standing	Not stated	Interview during postpartum	Preterm birth (22–36 weeks)	Prolonged standing [AOR 1.51(95% CI 0.97–2.35)], and long working hours [1.06 (95% CI 0.62–1.80)] were not associated with preterm birth	NS
								Heavy lifting [1.28 (1.17–2.57)], and high physical workload [AOR 2.31(95% CI 1.43–3.73)] were positively associated with preterm birth	Sig
				Lifting			Moderate preterm (33–36 weeks)	High physical workload was positively associated with moderate preterm [AOR 2.35(95% CI 1.41–3.94)]	Sig
				Working hours			Very preterm birth (22–32 weeks)	High physical workload was positively associated with very preterm birth [AOR 2.17(95% CI 1.01–4.65)]	Sig
				Physical			Spontaneous preterm birth	High physical workload was not associated with spontaneous preterm birth [AOR 1.74(95% CI 0.99–3.01]	NS
				workload			Medically indicated preterm birth	High physical workload was positively associated with indicated preterm birth [AOR 3.88 (95% CI 2.04–7.39)]	Sig
Henrich W et al. (2003) [34] Germany	1993	Case- control	707	Standing	Not stated	Interview during postpartum	Preterm birth (<37 weeks)	Prolonged standing was not associated preterm birth [COR 0.78 (*p* = 0.58)]	NS
Jansen PW et al. (2010) [35] Netherland	2002–2006	Prospective	4,408	Working hours	Not stated	Interview during pregnancy (postal questionnaire)	Preterm birth (<37 weeks)	Long working hours was not associated with preterm birth [AOR 1.30 (95% CI 0.81–2.10)]	NS
Kader et al. (2021) [36] Sweden	2008–2016	Prospective	4,970	Working hours Night Shift	1–12 weeks	Interview during pregnancy	Preterm birth (<37 weeks)	Long working hours [AOR 2.05 (95% CI 1.31–3.22)] during 3rd trimester was positively associated with preterm birth	Sig
					13–28 weeks			Long working hours during 1st trimester [AOR 0.77 (95% CI 0.47–1.25)] and 2nd trimester [AOR 1.04 (95% CI 0.64–1.69)] was not associated with preterm birth	
					29–42 weeks			High frequency night shift work during 1st trimester of pregnancy [AOR 1.62 (95% CI (1.03–2.53)] was	Sig
								positively associated with preterm birth but in 2nd trimester [AOR 1.26 (95% CI 0.79–2.00)], and 3rd trimester [AOR 0.61 (95% CI 0.29–1.25)] was not associated with preterm birth	NS
Knudsen et al (2017) [36] Denmark	1984–2010	Prospective	346,097	Lifting	Not stated	Interview during pregnancy	Preterm birth (22–37 weeks)	Heavy lifting was not associated with preterm birth [AOR 1.40 (95% CI 0.88–2.23)]	NS
Lawson et al. (2009) [49] USA	2001	Prospective	6,977	Standing	1st trimester of pregnancy	Interview during pregnancy (Mailed questionnaires)	Preterm birth (<37 weeks)	Prolonged standing [AOR 1.33 (95% CI 1.0–1.5)] during 1st trimester was positively associated with preterm birth	Sig
				Lifting Working Hours Shift work				Lifting [AOR 1.3 (95% CI 0.9–1.7)], long working hours [RR 1.2 (95% CI 0.8–1.2)], shift work [AOR 0.8 (95% CI 0.6–1.2)] were not associated with preterm birth	NS
Lee et al. (2017) [50] USA	1997–2009	Case- control	6,379	Physical workload	1st trimester	Interview during pregnancy	Preterm birth (<37 weeks)	Physical workload during the 1st trimester was positively associated with preterm birth [AOR 1.44 (95% CI 1.08–1.92)]	Sig
Magann et al. (2005) [51] USA	Not stated	Prospective	821	Standing	1st trimester	Interview during pregnancy	Preterm birth (20–37 weeks)	Prolonged standing [AOR 1.64 (95% CI 0.88–3.06)], and heavy lifting [AOR 1.14 (95% CI 0.32–3.18)] during 1st trimester were not associated with preterm birth	NS
				Lifting					
Mocevic et al. (2014) [38] Denmark	1996–2002	Prospective	65,530	Lifting	16 weeks	Job exposure matrix	Preterm birth (22–37 weeks)	Heavy lifting at 16th week was positively associated with preterm birth [AOR 1.22 (95% CI 1.05–1.42)]	Sig
							Moderate preterm birth (33–36 weeks)	Heavy lifting at 16th week was positively associated with moderate preterm birth [AOR1.19 (95% CI 1.01–1.40)]	Sig
							Very preterm birth (28–32 weeks)	Heavy lifting was not associated with very preterm birth. [AOR 1.53 (95% CI 0.98–2.37)]	NS
							Extremely preterm birth (22–27 weeks)	Heavy lifting was not associated with extremely preterm birth [AOR 0.88 (95% CI 0.26–2.95)]	NS
Nelson et al. (2009) [65] Thailand	2006–2007	Case- control	934	Physical workload	Not stated	Interview during post-partum	Preterm birth (22–36 weeks)	High physical workload during pregnancy was positively associated with preterm birth [AOR 2.42 (95% CI 1.15–5.09)]	Sig
							Moderate preterm birth (32–36 weeks)	High physical workload was not associated with moderate preterm birth [AOR 1.94 (95% CI 0.88–4.29)]	NS
							Very preterm birth (<32 weeks)	High physical workload was positively associated with very preterm birth [AOR 4.57 (95% CI 1.65–12.64)]	Sig
							Spontaneous preterm birth	High physical workload was not associated with spontaneous preterm birth [AOR 2.07 (95% CI 0.81–5.28)]	NS
							Medically indicated preterm	High physical workload was positively associated with medically indicated preterm birth [AOR 3.79 (95% CI 1.54–9.32)]	Sig
Niedhammer et al. (2009) [39] Ireland	2001	Prospective	1,124	Working hours	Not stated	Self-administered questionnaire and during pregnancy	Preterm birth (<37 weeks)	High physical workload [AOR 1.20 (95% CI 0.25–5.66)], long working hours [AOR 2.25 (95% CI 0.69–7.32)], shift work [1.68 (0.44–6.34)] were not associated with preterm birth	NS
				Shift work Physical workload					
Omokhodion et al. (2010) [62] Nigeria	2008	Cross-sectional	1,104	Physical workload	Not stated	Interview during post-partum	Preterm birth (<37 weeks)	High physical workload was not associated with preterm birth [AOR 1.52 (95% CI 0.97–2.39)]	NS
				Whole-body vibration				Whole-body vibration during pregnancy was positively associated with preterm birth [AOR 2.40 (95% CI 1.21–4.77)]	Sig
Pompeii et al. (2005) [52] USA	1995–2000	Prospective	1908	Standing	1–12 weeks	Telephone interview (during pregnancy)	Preterm birth (<37 weeks)	Prolonged standing during 1st trimester [AOR 1.2 (95% CI 0.9–1.7)], 2nd trimester [ 0.9 (95% CI 0.6–1.2), 3rd trimester of pregnancy [1.3 (95% CI 0.8–2.3)] was not associated with preterm birth Heavy lifting during 1st[AOR 1.3 (95% CI 0.9–1.8)], 2nd[AOR 1.3 (0.8–2.1)], 3rd [AOR 1.3 (95% CI 0.6–2.9)] trimester of pregnancy was not associated with preterm birth	NS
				Lifting					
				Night work	13–27 weeks			Long working hours during 1st [AOR 0.6 (95% CI 0.4–0.9) was negatively associated preterm birth	Sig^
				Working hours	28–31 weeks			Night work during 1st trimester [AOR 1.5 (95% CI 1.0–2.1)], and 2nd trimester [AOR 1.6 (95% CI (1.0–2.3)], was associated preterm birth	Sig
Rodrigues et al.(2008) [40] Portugal	Not stated	Case- control	1822	Working hours	Not stated	Interview during post-partum	Preterm birth (<37 weeks)	Prolonged standing [AOR 0.92 (95% CI 0.66–1.30)], physical workload [AOR 0.72 (95% CI 0.29–1.81)], long working hours [AOR 1.16 (95% CI 0.88–1.54)] during pregnancy were not associated with preterm birth	NS
				Standing					
				Physical workload					
Runge et al. (2013) [41] Denmark	1996–2002	Prospective	16 604	Lifting	Not stated	Telephone interviews (during pregnancy)	Moderate preterm birth (33–36 weeks)	Heavy lifting during pregnancy was not associated with moderate preterm birth [AOR 1.34 (95% CI 0.88–2.05)]	NS
							Very preterm birth (28–32 weeks)	Heavy lifting during pregnancy was not associated with very preterm birth [AOR 1.65 (95% CI 0.68–4.00)]	NS
							Extremely preterm (22–27 weeks)	Heavy lifting was statistically associated with extremely preterm [AOR (4.32 (95% CI 1.35–13.82)]	Sig
Saurel-Cubizolles et al. (2003) [42] European countries	1994–1997	Case- control	6,378	Working hours	1st trimester	Interview during post-partum	Preterm birth (22–36 weeks)	Prolonged standing [AOR 1.26 (95% CI 1.1–1.5)], and long working hours [AOR 1.33 (95% CI 1.1–1.6)] during 1st trimester of pregnancy were associated with preterm birth	Sig
				Standing				Heavy lifting [AOR 1.02 (95% CI 0.8–1.2)], shift work [AOR 0.97 (95% CI 0.8–1.1)], and during 1st trimester of pregnancy were not associated with preterm birth	NS
				Shift work Lifting					
Shirangi et al. (2009) [57] Australia	1960–2000	Retrospective	744	Working hours	Not stated	Mailed, self-administered questionnaire after birth	Preterm birth (22–37 Weeks)	Long working hours was associated with preterm birth [AHR 3.69 (95% CI 1.40–9.72)]	Sig
Skroder et al. (2021) [43] Sweden	1994–2014	Prospective	527,359	Whole body vibration	Not stated	Job-exposure matrix	Preterm birth (<37 weeks)	Whole-body vibration was not associated with preterm birth [AOR 1.36 (1.01–1.84)]	Sig
Snijder et al. (2012) [44] Netherlands	2002–2006	Prospective	4,680	Standing Lifting Working hours Shiftwork	20 weeks	Interview during pregnancy	Preterm birth (<37 weeks)	Prolonged standing [AOR 1.03 (AOR CI 0.72–1.46)], heavy lifting [AOR 0.58 (95% CI 0.14–2.39)], shift work [AOR 1.41 (95% CI 0.51–3.92)] during 2nd trimester of pregnancy was not associated with preterm birth	NS
					30 weeks			Long working hours [AOR 1.58 (95% CI 1.06–2.35)] during 2nd trimester of pregnancy was associated with preterm birth	Sig
Specht et al (2019) [45] Denmark	2007–2015	Prospective	16,501	Night work	1–22 weeks	Payroll record	Preterm birth (23–37 weeks)	Night work during 1st trimester [AOR 1.31(95% CI 1.06–1.61)], and 2nd trimester of pregnancy [AOR 1.30 (95% CI 1.02–1.66)] was associated with preterm birth	Sig
(Stinson et al. 2003) [53] USA	Not stated	Prospective	359	Night work	22–26 weeks	Interview during pregnancy	Preterm birth (<37 weeks)	Night work during 2nd trimester of pregnancy was not associated with preterm birth [COR = 0.36, *p* = 0.234]	NS
Sumsrisuwan et al. (2015) [66] Thailand	2013–2014	Retrospective	572	Rotating shift work	Not stated	(Self-administered questionnaire) during post-partum	Preterm birth (<37 weeks)	Shift work was positively associated with preterm birth [AOR 3.64 (95% CI 1.33–9.95)]	Sig
Takeuchi et al. (2014) [56] Japan	2009–2011	Retrospective	939	Working hours	1st trimester	Self-administered survey during post-partum	Preterm birth (<37 weeks)	Long working hours during 1st trimester of pregnancy was associated with preterm birth [AOR 2.46 (95% CI 1.16–5.23)]	Sig
Von Ehrenstein et al. (2014) [54] USA	2003	Case-control	1,341	Physical workload	Not stated	Job exposure matrix	Preterm birth (<37 weeks)	Physical workload during 1st trimester of pregnancy [AOR 1.40 (95% CI 0.95–2.06)] was not associated with preterm birth	NS
				Shiftwork				Shift work [AOR3.52 (95% CI 1.36–9.14)] was associated with preterm birth	Sig
Vrijkotte et al. (2021) [46] Netherlands	2003–2004	Prospective	4,865	Standing Physical workload Working hours	1st trimester	Interview during pregnancy	Preterm birth (24–37 weeks)	Prolonged standing during 1st trimester of pregnancy [1.80 (95% CI 1.19–2.74)] was associated with preterm birth	Sig
								High physical workload during 1st trimester of pregnancy [AOR 1.15 (95% CI 0.67–3.95)], and long working hours [AOR 1.18 (95% CI 0.78–1.81)] were not associated with preterm birth	NS
							Spontaneous preterm birth	Prolonged standing [AOR 1.30 (95% CI 0.78–2.16)], long working hours [AOR 0.95 (95% CI 0.51–1.78)], and physical workload [AOR 0.81 (95% CI 0.48–1.37)] were not associated with spontaneous preterm birth	NS
							Medically indicated preterm birth	Prolonged standing during pregnancy [AOR 2.09 (95% CI 1.00–4.97)]] was associated with preterm birth	Sig
								Long working hours [1.15 (95% CI 0.37–3.55)], and physical workload [AOR1.68 (95% CI 0.67–4.22)] were not associated with medically indicated preterm birth	NS
Zhu et al. (2004) [47] Denmark	1998–2001	Prospective	1,699	Shift work	11–25 weeks	Telephone interview during pregnancy	Preterm birth (<37 weeks)	Shift work during 1st and 2nd trimester of pregnancy [AOR 0.82 (95% CI 0.61–1.11)] was not associated with preterm birth	NS
					27–37 weeks				

#### Study Design

Of the included studies, twenty-one studies were prospective [30–32, 35–39, 41, 43–49, 51–53, 55, 63], nine studies were case control [33, 34, 40, 42, 50, 54, 58, 59, 65], three studies were retrospective [56, 57, 66], and four cross-sectional studies [60–62, 64]. In 21 cohort investigations [30–32, 35–39, 41, 43–49, 51–53, 55, 63] exposure was ascertained prospectively during pregnancy, whereas for 16 studies [33, 34, 40, 42, 50, 54, 56–62, 64–66] (nine case-control, three retrospective cohort, and four cross-sectional studies), information about exposure was elicited after the relevant birth outcome had occurred.

#### Exposure Assessment and Sample Size

The data on exposure were collected mostly through self-report (by telephone or interview and mail), but in some studies job title was used as surrogate index of exposure [31, 38, 43, 45, 48, 54]. Of the included studies, 19 examined a single exposure [31, 32, 34, 35, 37, 38, 41, 43, 45, 47, 48, 50, 53, 56, 57, 63–66], six examined two exposures [36, 51, 54, 59, 60, 62], five examined three exposures [39, 40, 46, 55, 61], six examined four exposures [31, 33, 42, 44, 49, 52], and one examined five exposures [58]. Eight studies also reported the time of exposure as being during the 1^st^ trimester, three studies at 2^nd^ trimester, one study at 3^rd^ trimester, five studies at all trimester, one study both at 2^nd^ and 3^rd^ trimester and the remaining 20 studies did not state the exposure timing by trimester. The included studies involved 1,054,008 participants with sample size ranging from 127 to 527,359 participants [43, 55].

#### Outcome

Except for two studies, preterm birth was determined using hospital records, registers, or birth certificates [55, 66]. All but nine of the studies used the World Health Organization’s definition of preterm birth, which is the birth of a live fetus before 37 completed weeks of pregnancy [33, 37, 38, 41, 42, 45, 46, 57, 65].

#### Methodological Risk of Bias Assessment

Methodological risk of bias assessment was conducted on thirty-seven studies, 27 were classified as having low-risk of bias [30–33, 35–38, 41–46, 48, 50–52, 56–59, 61, 62, 64–66], two were classified as having moderate risk of bias [54, 55], and eight were classified as having high risk of bias [34, 39, 40, 47, 49, 53, 60, 63] (See Supplementary Material S2).

#### Potential Cofounding Factors

Thirty-two studies controlled for potential confounding factors using various methods, including matching, restriction, stratification, and multivariate regression modeling. However, five studies did not address confounding at all [34, 53, 59, 60, 63]. Of the 32 studies including statistical adjustment for confounding, maternal age was the most commonly adjusted for variable, in 29 studies [32, 33, 35–52, 54–58, 61, 62, 65, 66] followed by maternal education (*n* = 22 studies) [30–33, 35, 36, 40–44, 46, 48–52, 54, 58, 61, 62, 65], parity (number of live births) (*n* = 20 studies) [30–32, 35–37, 39–41, 44–46, 48, 49, 52, 56, 58, 61, 62, 65], maternal smoking (*n* = 15 studies) [35–39, 42–47, 50, 52, 56] and hypertension during pregnancy [33, 42, 43, 49, 50, 54, 58, 62] (*n* = 8 studies). Of the five studies not using statistical adjustment, four used the Chi-square test to examine association between exposure and outcome [34, 53, 55, 60].

#### Certainty Assessment (GRADE)

The overall certainty of evidence ranged from very low to moderate for each of the six exposure categories (See Supplementary Table S3). All the included studies were observational studies, and thus started as low-certainty assessments. The most common reasons for downgrading the certainty of evidence were [1] indirectness [2], imprecision and [3] inconsistency (*n* = 1). On the other hand, the most common reason for uprating certainty was large effect size and adjustment for plausible cofounding. Although observational studies started as low certainty evidence, we found a moderately certain evidence for the exposure categories physical workload, working hours, shift work, whole-body vibration, which were rated up. On the other hand, due to indirectness and impression, the certainty of evidence was downgraded into very-low evidence for the exposure categories prolonged standing and heavy lifting. There was no evidence of publication bias within the included studies.

### The Relation Between Physical Occupational Risks and Preterm Birth

#### Physical Workload

Ten of the included studies investigated the relationship between physical workload and preterm birth [33, 39, 40, 46, 48, 50, 54, 61, 62, 65]. Two studies with a higher risk of bias were excluded from further analysis [39, 40]. Of the remaining eight high-quality studies, six found a statistically significant positive association between physical workload and preterm birth [33, 48, 50, 54, 61, 65], while the other two did not find such a relationship [46, 62]. Overall, there is moderate evidence that physical workload is associated with an increased risk of preterm birth. However, due to differences in how physical workload was measured across the studies, it was not possible to calculate a precise estimate of the effect of physical workload on preterm birth.

#### Working Hours

**FIGURE 2:**
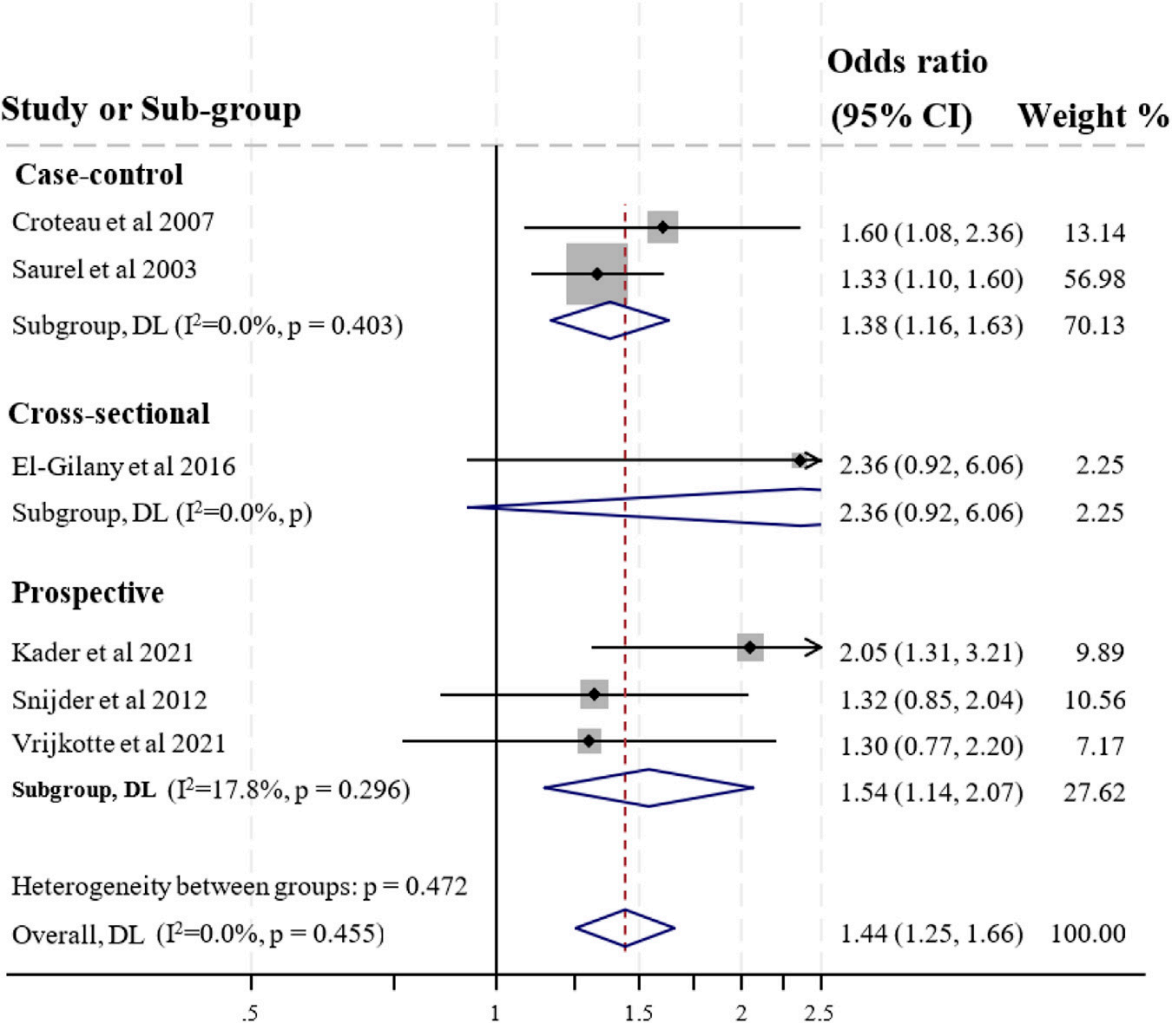
Forest plot for preterm birth and working >40 h per week during pregnancy (Australia, 2023).

Sixteen studies analysed the relationship between long working hours and preterm birth [30, 33, 35, 36, 39, 40, 42, 44, 46, 49, 52, 55–58, 61]. Three studies had high ROB, and thus were excluded in further synthesis and meta-analysis [39, 40, 49]. Six low ROB [36, 42, 55–58, 61] and one moderate ROB studies [55] reported a positive statistically significant association between long working hours and preterm birth. One study found a negative relationship [52] and five studies showed no statistical association between working hours and preterm birth [30, 33, 35, 44, 46]. The overall finding was moderate evidence of a positive association between long working hours and preterm birth. Six low ROB studies were feasible to combine in formal meta-analysis on the relationship between working hours (>40 h/day vs. less) and preterm birth. The pooled effect estimate based on four studies was 1.44 (1.25–1.66) (see Figure 2).

#### Shift Work

**FIGURE 3:**
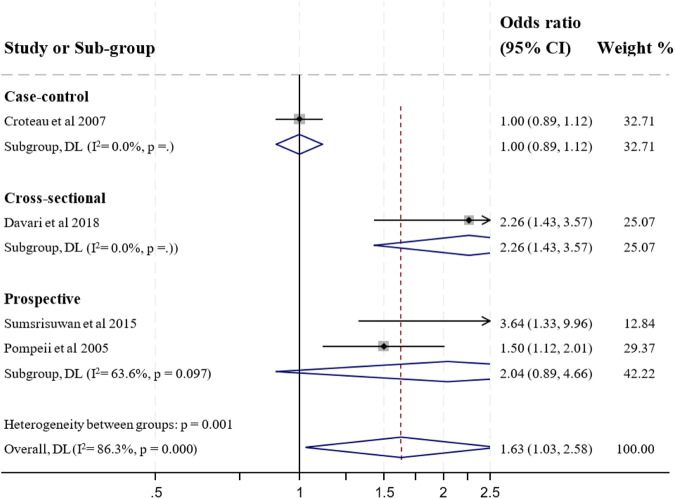
Forest plot for preterm birth and shift work during pregnancy (Australia, 2023).

The relationship between shiftwork and pre-term birth was examined in fifteen studies [31, 36, 39, 44, 45, 47, 49, 52–55, 58, 60, 64, 66]. Five studies with high ROB were excluded from further synthesis and meta-analysis [39, 47, 49, 53, 60]. The remaining four studies with low ROB [36, 52, 64, 66] and two study with moderate ROB [54, 55] showed a positive relationship between shift work and preterm birth. One study showed that working night shift in the third trimester of pregnancy was protective for the occurrence of preterm birth [31]. Three studies reported no association between shift work and preterm birth [44, 45, 58]. Hence, the overall result showed a moderate evidence of a positive statistically significant association between shift work and preterm birth. Of ten studies, four studies with low ROB were feasible to include in a formal meta-analysis on the relationship between shift work or night work (Yes vs. No) and preterm birth. The pooled effect estimate based on four studies was 1.63 (1.03–2.58) (see Figure 3).

#### Whole-Body Vibration (WBV)

The relationship between whole-body vibration and preterm birth was assessed in three studies all of which were rated as having low ROB [43, 58, 62]. All of these studies reported a positive statistically significant association between whole-body vibration and preterm birth [43, 58, 62]. The overall finding showed moderate evidence of a positive statistical association between whole-body vibration and increased odds of preterm birth. Due to exposure definition differences, meta-analysis was not possible.

#### Standing

Of the included studies, fourteen studies examined the relationship between standing and preterm-birth [30, 32–34, 42, 44, 46, 49, 51, 52, 55, 58, 60, 63]. Four studies had high risk of bias and thus were excluded from further synthesis and meta-analysis [34, 49, 60, 63]. Of the included studies for further synthesis, two low ROB studies [30, 46] and one moderate ROB study described a positive statistically significant relationship between prolonged standing and pre-term birth [55]. The remaining seven low ROB studies did not find a statistically significant relationship between prolonged standing and preterm birth [30, 32, 33, 44, 51, 52, 58]. Overall these findings indicate very low evidence of no statistically significant association between prolonged standing and preterm birth. Because of discrepancies in defining exposure, conducting a meta-analysis for prolonged standing and preterm birth was impossible.

#### Lifting

Twelve studies examined the relationship between lifting and preterm birth [30, 33, 37, 38, 42, 44, 49, 51, 52, 58, 59, 61], of which 11 studies had low risk of bias [30, 33, 37, 38, 42, 44, 51, 52, 58, 59, 61]. One study had high risk of bias and thus was excluded from further synthesis [49]. Four of the elven included studies found a positive statistically significant relationship between lifting and preterm birth [33, 38, 59, 61]. The remaining seven studies did not find a statistically significant association between lifting and preterm birth [30, 37, 42, 44, 51, 52, 58]. Overall, findings indicated very low evidence of no statistically significant association between heavy lifting and preterm birth. Due to disparities in the definition of exposure, conducting a meta-analysis for heavy lifting and preterm birth was rendered infeasible.

#### Secondary Outcomes: Type of Pre-term Birth

Three low ROB studies examined the relationship between physical workload and medically indicated preterm birth and/or spontaneous preterm birth [33, 46, 65]. Two of the three studies reported a positive statistically significant association between high physical workload and medically indicated preterm birth [33, 65], suggesting moderate evidence of a relationship. However, all three studies reported no statistical association between high physical workload and spontaneous preterm birth [33, 46, 65], providing moderate evidence of no association. Two low ROB studies examined the relationship between physical workload and very preterm birth or moderate preterm birth [33, 65]. Both reported a positive statistically significant association with very pre-term birth providing moderate evidence of an association [33, 65]. One study showed a positive association between high physical workload and moderate preterm birth, providing inconclusive evidence of a relationship [33]. Two low ROB studies investigated the relationship between heavy lifting and moderate preterm birth, very preterm birth [38, 41], and extremely preterm birth. Both reported no association between heavy lifting and moderate preterm birth and very preterm birth [38, 41]. However, a single study reported a positive statistical association between heavy lifting and extremely preterm birth, providing inconclusive evidence [41].

## Discussion

A systematic review and meta-analysis found that physical occupational risk factors during pregnancy are associated with an increased risk of preterm birth. Preterm birth is a serious pregnancy complication linked to long-term neurodevelopmental problems and chronic health conditions in children [67, 68]. This review found moderate evidence that high physical workload, long working hours, shift work, and whole-body vibration during pregnancy increase the risk of preterm birth. It also found that high physical workload may contribute to medically indicated and very preterm birth. However, there are gaps in the evidence base on the association of physical occupational risks and preterm birth, suggesting opportunities for future research.

Although it is challenging to demonstrate a causal relationship between physical occupational exposures and adverse perinatal outcomes (preterm birth) due to the observational nature of these studies, there are plausible potential physiological mechanisms for this association. These include that high physical workload, long working hours, shift work and whole-body vibration may cause fatigue [69], stress, sleep deprivation, and circadian rhythm disruption [70, 71], this result increased release of catecholamine [72], increased prostaglandins production [73] and corticosterone level [74] which may increase uterine contractility and decrease placental function [75]. This could in turn lead to preterm birth. It could also be that women who work in physically demanding jobs, long working hours, shift work, and whole-body vibrations are also exposed to other occupational risks, social, psychological, life style or environmental risk factors for pre-term birth that are not accounted for in these observational studies (i.e., unobserved confounding) [76–79]. For example women in physically demanding jobs may also have lower incomes than those in “white collar” jobs (professional, office-based, or administrative occupations), which may affect multiple determinants of maternal and neonatal health such as nutrition and access to healthcare [80]. Some studies in this review took socioeconomic factors into account, but most did not consider other common occupational risks that may be interconnected. It is important to comprehensively understand how these occupational risks, such as psychosocial work factors, can contribute to preterm birth. This finding suggests that preterm birth may be preventable in some working women by reducing their exposure to heavy physical workloads, long working hours, shift work, and whole-body vibrations. Pregnant women should be aware of the risks associated with these occupational risks and take steps to minimize their exposure. Employers and regulatory authorities have a responsibility to create policies and work practices that reduce the exposure of pregnant women to these hazards.

This systematic review also identified moderate evidence of a positive association between high physical workload and medically indicated and very-preterm birth [33, 65]. There may be biological mediators that explain this relationship like the presence of hypertriton during pregnancy [81]. For example, women in the Canada who experienced physical workload and pre-eclampsia had greater risk of medically indicated preterm birth and very preterm birth [82, 83]. Hence, high physical demanding jobs potentially increases the risk of or pre-eclampsia and more likely to have a medically indicated preterm birth. The results indicate that a need to separate preterm births into subcategories to properly evaluate the relationship between high physical workload and preterm births.

In this systematic review we found a large number of studies on the relationship between physical occupational risks and preterm birth from developed countries and very few studies from low-income countries [48, 55, 56, 59–62, 64–66] though there are many babies born preterm in these regions (9.3% vs. 12%) respectively [68]. Female labor force participation is notably high in both low-income and high-income countries worldwide, with significant shifts in job characteristics over the past decades [84]. Similarly, substantial progress has been achieved in maternal and child healthcare services in recent decades, although maternal and neonatal mortality rates continue to remain high [85]. Majority of the included studies had collated data and published before 2000 and 2013 respectively [30–35, 39, 40, 42, 44, 47–49, 51–53, 57–60, 62, 63, 65]. There is a lack of recent evidence on how the changing nature of jobs and occupational exposures affect pregnant women and their babies. Researchers need to study the link between occupational exposures (such as psychosocial job strain, working hours, and shift work) and preterm birth. There is also a need for employers to consider modifying the physical working environment and working conditions for pregnant women to reduce the risk of preterm birth and other negative birth outcomes.

### Strengths and Limitations of This Review

This review’s strength lies in its rigorous methodology, including risk assessment and GRADE synthesis. It uniquely focuses on working pregnant women, avoiding potential bias introduced by comparing them with unemployed individuals. This approach ensures greater relevance to the target audience and enhances the review’s credibility [86, 87]. To minimize bias, this review exclusively considered studies involving employed women in both exposure and control groups. However, it has limitations, including the restriction to English-language articles, potentially missing studies in other languages. Additionally, reliance on data solely from observational studies increased result heterogeneity and reduced evidence certainty. Most studies assessed occupational physical exposures through self-reported measures, potentially introducing recall bias.

## Conclusion

This systematic review and meta-analysis found that working in physically demanding jobs, long hours, shift work, and jobs that expose women to whole-body vibration increase the chance of having preterm birth. Further research is needed to investigate the effect of occupational risks on preterm birth among employed pregnant women, using a follow-up design and evidence synthesis.
