# Supplementary material for: Maternal Occupational Risk Factors and Preterm Birth: A Systematic Review and Meta-Analysis
Source: Public Health Rev. 2023 Oct 23;44:1606085. doi: 10.3389/phrs.2023.1606085 (PMC10625911; doi:10.3389/phrs.2023.1606085)
Supplement: Supplementary file 1 [file Table1.DOCX]

**Table 1: Draft of search strategy to be used using Medline electronic database.**

| **Number** | **Search terms** | **Results from 19 Aug 2022** |
| --- | --- | --- |
| 1 | Occupational Exposure/ | 59,544 |
| 2 | Lifting/ or Workload/ or employment/ or standing position/ | 75,767 |
| 3 | Shift Work Schedule/ or Work Schedule Tolerance/ | 8,055 |
| 4 | “Occupational Exposure*”.mp. | 68,100 |
| 5 | (occupational activit* or workload).mp. | 44,193 |
| 6 | ((activ* adj2 (intens* or physical* or vigor*)) and (work* or occupation*)).mp. | 18,518 |
| 7 | (heavy exertion* or heavy lifting or heavy work*).mp. | 1,909 |
| 8 | (((demand* or drain* or exhaust* or fatigu*) adj2 physical*) and (work* or occupation*)).mp. | 2,768 |
| 9 | (prolonged adj5 (standing or walking or bending or upright)).mp. | 808 |
| 10 | (long work* hour* or long work* day*).mp. | 981 |
| 11 | ''whole-body vibration''.mp. | 1,958 |
| 12 | working time.mp. | 2,319 |
| 13 | (shift work or afternoon shift or evening shift or morning shift or night shift or rotating shift or shift schedule).mp. | 6,262 |
| 14 | 1 or 2 or 3 or 4 or 5 or 6 or 7 or 8 or 9 or 10 or 11 or 12 or 13 | 194,240 |
| 15 | Premature Birth/ | 18,969 |
| 16 | infant, premature/ or infant, extremely premature/ | 62,785 |
| 17 | Obstetric Labor, Premature/ | 13,703 |
| 18 | ((preterm or pre-term or premature) adj (birth* or infan* or babby or babies or neonat* or labo?r or deliver*)).mp. | 95,610 |
| 19 | extreme* prematur*.mp. | 5,125 |
| 20 | 15 or 16 or 17 or 18 or 19 | 123,290 |
| 21 | 14 and 20 | 522 |
| 22 | pregnancy outcome/ or pregnancy complication/ | 56,159 |
| 23 | (“adverse pregnancy outcome” or “adverse birth outcome”).mp. | 1,887 |
| 24 | 22 or 23 | 56,793 |
| 25 | 14 and 24 | 485 |
| 26 | 21 or 25 | 863 |
| **27** | **limit 26 to yr="2000 -Current"** | **569** |

**Table 2: Draft of search strategy to be used using Embase electronic database.**

| **Number** | **Search terms** | **Results from 19 Aug 2022** |
| --- | --- | --- |
| 1 | Occupational Exposure/ or whole-body vibration/ | 89,761 |
| 2 | Lifting/ or Workload/ or standing/ or erect posture/ | 194,353 |
| 3 | Shift Work Schedule/ or Work Schedule Tolerance/ or Working time/ or night work/ | 21,818 |
| 4 | “Occupational Exposure”.mp. | 95,696 |
| 5 | (occupational activit* or workload).mp. | 71,659 |
| 6 | ((activ* adj2 (intens* or physical* or vigor*)) and (work* or occupation*)).mp. | 39,781 |
| 7 | (heavy exertion* or heavy lifting or heavy work*).mp. | 3,327 |
| 8 | (((demand* or drain* or exhaust* or fatigu*) adj2 physical*) and (work* or occupation*)).mp. | 4,336 |
| 9 | (prolonged adj5 (standing or walking or bending or upright)).mp. | 1,422 |
| 10 | (long work* hour* or long work* day*).mp. | 1,401 |
| 11 | ''whole-body vibration''.mp. | 3,229 |
| 12 | working time.mp. | 13,134 |
| 13 | (shift work or afternoon shift or evening shift or morning shift or night shift or rotating shift or shift schedule).mp. | 12,113 |
| 14 | 1 or 2 or 3 or 4 or 5 or 6 or 7 or 8 or 9 or 10 or 11 or 12 or 13 | 377,022 |
| 15 | Premature Birth/ | 84,555 |
| 16 | infant, premature/ or infant, extremely premature/ | 131,442 |
| 17 | Obstetric Labor, Premature/ | 27,044 |
| 18 | ((preterm or pre-term or premature) adj (birth* or infan* or babby or babies or neonat* or labo?r or deliver*)).mp. | 159,206 |
| 19 | extreme* prematur*.mp. | 3,429 |
| 20 | 15 or 16 or 17 or 18 or 19 | 208,677 |
| 21 | 14 and 20 | 949 |
| 22 | pregnancy outcome/ or birth outcome/ | 73,866 |
| 23 | ("adverse pregnancy outcome" or "adverse birth outcome").mp | 3,055 |
| 24 | 22 or 23 | 74,853 |
| 25 | 14 and 24 | 556 |
| 26 | 21 or 25 | 1,326 |
| **27** | **limit 26 to yr="2000 -Current"** | **1,122** |

**Table 3: Draft of search strategy to be used using Emcare electronic database.**

| **Number** | **Search terms** | **Results from 19 Aug 2022** |
| --- | --- | --- |
| 1 | Occupational Exposure/ or Occupational hazard/ or Occupational health/ or whole-body vibration/ | 42,237 |
| 2 | Lifting/ or Workload/ or standing/ or erect posture/ or Employment/ | 112,898 |
| 3 | Shift Work Schedule/ or Work Schedule Tolerance/ or Working time/ or night work/ | 9508 |
| 4 | “Occupational Exposure”.mp. | 21,238 |
| 5 | (occupational activit* or workload).mp. | 26,927 |
| 6 | ((activ* adj2 (intens* or physical* or vigor*)) and (work* or occupation*)).mp. | 11,794 |
| 7 | (heavy exertion* or heavy lifting or heavy work*).mp. | 1,233 |
| 8 | (((demand* or drain* or exhaust* or fatigu*) adj2 physical*) and (work* or occupation*)).mp. | 2,071 |
| 9 | (prolonged adj5 (standing or walking or bending or upright)).mp. | 504 |
| 10 | (long work* hour* or long work* day*).mp. | 651 |
| 11 | ''whole-body vibration''.mp. | 1,700 |
| 12 | working time.mp. | 6,029 |
| 13 | (shift work or afternoon shift or evening shift or morning shift or night shift or rotating shift or shift schedule).mp. | 3,996 |
| 14 | 1 or 2 or 3 or 4 or 5 or 6 or 7 or 8 or 9 or 10 or 11 or 12 or 13 | 182,742 |
| 15 | Premature Birth/ | 28,029 |
| 16 | infant, premature/ or infant, extremely premature/ | 41,552 |
| 17 | Obstetric Labor, Premature/ | 10,508 |
| 18 | ((preterm or pre-term or premature) adj (birth* or infan* or babby or babies or neonat* or labo?r or deliver*)).mp. | 47,240 |
| 19 | extreme* prematur*.mp. | 1,291 |
| 20 | 15 or 16 or 17 or 18 or 19 | 63,714 |
| 21 | 14 and 20 | 596 |
| 22 | pregnancy outcome/ or pegnancy complication/ | 21,175 |
| 23 | ((pregnanc* or birth*) adj (outcom* or complicatio*)).mp. | 940 |
| 24 | 22 or 23 | 21,562 |
| 25 | 14 and 24 | 304 |
| 26 | 21 or 25 | 796 |
| **27** | **limit 26 to yr="2000 -Current"** | **656** |

**Table 4: Draft of search strategy to be used using CINHAL electronic database.**

| **Number** | **Search terms** | **Results from 19 Aug 2022** |
| --- | --- | --- |
| 1 | (MH "Occupational Exposure") | 20,697 |
| 2 | (MH "Occupational Hazards") | 6,756 |
| 3 | (MH "Occupational Health") | 29,932 |
| 4 | (MH "Lifting") | 2,870 |
| 5 | (MH "Workload") | 17,803 |
| 6 | (MH "Standing") | 2,578 |
| 7 | (MH "Employment") | 24,311 |
| 8 | (MH "Shiftwork") | 4,470 |
| 9 | "Occupational Exposure" | 21,758 |
| 10 | ("occupational activit*" or workload) | 28,924 |
| 11 | ((activ* W2 (intens* or physical* or vigor*)) and (work* or occupation*)) | 981 |
| 12 | ("heavy exertion*" or "heavy lifting" or "heavy work*") | 1,060 |
| 13 | (((demand* or drain* or exhaust* or fatigu*) W2 physical*) and (work* or occupation*)) | 362 |
| 14 | (prolonged W5 (standing or walking or bending or upright)) | 364 |
| 15 | ("long work*" hour* or "long work*" day*) | 496 |
| 16 | ''whole-body vibration'' | 1,178 |
| 17 | "working time" | 808 |
| 18 | ("shift work" or "afternoon shift" or "evening shift" or "morning shift" or "night shift" or "rotating shift" or "shift schedule") | 2,985 |
| 19 | S1 OR S2 OR S3 OR S4 OR S5 OR S6 OR S7 OR S8 OR S9 OR S10 OR S11 OR S12 OR S13 OR S14 OR S15 OR S16 OR S17 OR S18 | 118,015 |
| 20 | (MH "Childbirth, Premature") | 12,699 |
| 21 | (MH "Infant, Premature") | 26,263 |
| 22 | (MH "Labor, Premature") | 3,632 |
| 23 | ((preterm or pre-term or premature) W1(birth* or infan* or babby or babies or neonat* or labo?r or deliver*)) | 38,121 |
| 24 | extreme* prematur* | 1,081 |
| 25 | S20 OR S21 OR S22 OR S23 OR S24 | 53,184 |
| 26 | S19 AND S25 | 234 |
| 27 | (MH "Pregnancy Outcomes") | 27,366 |
| 28 | MH "Pregnancy Complications") | 21,569 |
| 29 | ((pregnanc* or birth*) W1 (outcom* or complicatio*)) | 63,021 |
| 30 | S27 OR S28 OR S29 | 63,021 |
| 31 | S19 AND S30 | 450 |
| 32 | S26 OR S31 | 603 |
| **33** | **limit 32 to yr="2000 -Current"** | **503** |

**Table 5: Draft of search strategy to be used using Scopus electronic database.**

| **Number** | **Search terms** | **Results from 19 Aug 2022** |
| --- | --- | --- |
| 1 | “Occupational Exposure” | 109,442 |
| 2 | (“occupational activit*” or workload) | 145,119 |
| 3 | ((activ* W/2 (intens* or physical* or vigor*)) and (work* or occupation*)) | 44,643 |
| 4 | (“heavy exertion*” or “heavy lifting” or “heavy work*”) | 5,414 |
| 5 | (((demand* or drain* or exhaust* or fatigu*) W/2 physical*) and (work* or occupation*)) | 6,185 |
| 6 | (prolonged W/2 (standing or walking or bending or upright)) | 1,224 |
| 7 | (“long work*” hour* or “long work*” day*) | 702 |
| 8 | “whole-body vibration” | 4,612 |
| 8 | “working time” | 17,565 |
| 9 | (“shift work” or “afternoon shift” or “evening shift” or “morning shift” or “night shift” or “rotating shift” or “shift schedule”) | 12,828 |
| 10 | 1 or 2 or 3 or 4 or 5 or 6 or 7 or 8 or 9 or 10 or 11 or 12 or 13 | 328,922 |
| 11 | ((preterm or “pre-term” or premature) W/2 (birth* or infan* or babby or babies or neonat* or labo?r or deliver*)) | 149,733 |
| 12 | extreme* prematur* | 15,849 |
| 13 | 11 or 112 | 154,570 |
| 14 | 10 and 13 | 626 |
| **19** | **limit 26 to yr="2000 -Current"** | 487 |

**Table 6: Draft of search strategy to be used using Web of Science electronic database.**

| **Number** | **Search terms** | **Results from 19 Aug 2022** |
| --- | --- | --- |
| 1 | “Occupational Exposure” | 26,510 |
| 2 | (“occupational activit*” or workload) | 86,602 |
| 3 | ((activ* NEAR/2 (intens* or physical* or vigor*)) and (work* or occupation*)) | 36,867 |
| 4 | (“heavy exertion*” or “heavy lifting” or “heavy work*”) | 3,313 |
| 5 | (((demand* or drain* or exhaust* or fatigu*) NEAR/2 physical*) and (work* or occupation*)) | 4,836 |
| 6 | (prolonged NEAR/2 (standing or walking or bending or upright)) | 1,091 |
| 7 | (“long work*” hour* or “long work*” day*) | 1,847 |
| 8 | “whole-body vibration” | 4,739 |
| 8 | “working time” | 6,432 |
| 9 | (“shift work” or “afternoon shift” or “evening shift” or “morning shift” or “night shift” or “rotating shift” or “shift schedule”) | 10,246 |
| 10 | 1 or 2 or 3 or 4 or 5 or 6 or 7 or 8 or 9 or 10 | 174,194 |
| 11 | ((preterm or “pre-term” or premature) NEAR/2 (birth* or infan* or babby or babies or neonat* or labo?r or deliver*)) | 120,596 |
| 12 | extreme* prematur* | 8,274 |
| 13 | “adverse pregnancy outcome” | 2,026 |
| 13 | 11 or 112 or 13 | 125,036 |
| 14 | 10 and 13 | 445 |
| **19** | **limit 26 to yr="2000 -Current"** | 375 |
