# Supplementary material for: Maternal Occupational Risk Factors and Preterm Birth: A Systematic Review and Meta-Analysis
Source: Public Health Rev. 2023 Oct 23;44:1606085. doi: 10.3389/phrs.2023.1606085 (PMC10625911; doi:10.3389/phrs.2023.1606085)
Supplement: Supplementary file 2 [file Table2.docx]

|  |  | **Summary of the design-specific critical appraisal results** | | | | | |  |  |  |  |  |  |
| --- | --- | --- | --- | --- | --- | --- | --- | --- | --- | --- | --- | --- | --- |
| **Study design** | **Critical appraisal questions and their responses** | | |  |  |  |  |  |  |  |  |  |  |
| **Analytical cross-sectional study** | |  |  |  |  |  |  |  |  |  |  |  |  |
| **Author and Year** | Were the criteria for inclusion in the sample clearly defined? (Q1) | Were the study subjects and the setting described in detail?(Q2) | Was the exposure measured in a valid and reliable way?(Q3) | Were objective, standard criteria used for measurement of the condition? (Q4) | Were confounding factors identified? (Q5) | Were strategies to deal with confounding factors stated? (Q6) | Were the outcomes measured in a valid and reliable way? (Q7) | Was appropriate statistical analysis used? (Q8) |  |  |  | Total (%) | Overall risk of bias |
| Arafa et al. (2007) | Yes | Yes | No | No | No | No | Yes | No |  |  |  | 37.5 | High |
| Davari et al. (2018) | Yes | Yes | Yes | No | Yes | Yes | Yes | Yes |  |  |  | 87.5 | Low |
| El-Gilany et al. (2016) | Yes | Yes | Yes | No | Yes | Yes | Yes | Yes |  |  |  | 87.5 | Low |
| Sumsrisuwan et al. (2015) | Yes | Yes | Yes | No | Yes | Yes | Yes | Yes |  |  |  | 87.5 | Low |
| Omokhodion et al. (2010) | Yes | Yes | No | Yes | Yes | Yes | Yes | Yes |  |  |  | 87.5 | Low |
| **Case-control study** |  |  |  |  |  |  |  |  |  |  |  |  |  |
| Author and Year | Were the groups comparable other than the presence of disease in cases or the absence of disease in controls? (Q1) | Were cases and controls matched appropriately? (Q2) | Were the same criteria used for identification of cases and controls? (Q3) | Was exposure measured in a standard, valid and reliable way? (Q4) | Was exposure measured in the same way for cases and controls? (Q5) | Were confounding factors identified? (Q6) | Were strategies to deal with confounding factors stated? (Q7) | Were outcomes assessed in a standard, valid and reliable way for cases and controls? (Q8) | Was the exposure period of interest long enough to be meaningful? (Q9) | Was appropriate statistical analysis used? (Q10) |  |  |  |
| Agbla et al. (2006) | Yes | No | Yes | Yes | Yes | Yes | No | Yes | Yes | Yes |  | 80 | Low |
| Croteau et al. (2007) | Yes | No | Yes | Yes | Yes | Yes | Yes | Yes | Yes | Yes |  | 90 | Low |
| Escribà-Agüir et al. (2001) | Yes | No | Yes | Yes | Yes | Yes | No | Yes | Yes | Yes |  | 80 | Low |
| Henrich W et al. (2003) | No | Yes | No | No | Yes | No | No | No | Yes | No |  | 30 | High |
| Nelson et al. (2009) | Yes | No | Yes | Yes | Yes | Yes | No | Yes | Yes | Yes |  | 80 | Low |
| Rodrigues et al.(2008) | No | No | Yes | No | Yes | Yes | No | No | No | Yes |  | 40 | High |
| Saurel-Cubizolles et al. (2003) | Yes | No | Yes | No | Yes | Yes | Yes | Yes | Yes | Yes |  | 80 | Low |
| Von Ehrenstein et al. (2014) | Yes | Yes | No | Yes | No | Yes | No | Yes | Yes | Yes |  | 63.6 | Moderate |
| **Cohort Study** |  |  |  |  |  |  |  |  |  |  |  |  |  |
| Author and Year | Were the two groups similar and recruited from the same population?(Q1) | Were the exposures measured similarly to assign people to both exposed and unexposed groups? (Q2) | Was the exposure measured in a valid and reliable way? (Q3) | Were confounding factors identified? (Q4) | Were strategies to deal with confounding factors stated? (Q5) | Were the groups/participants free of the outcome at the start of the study (or at the moment of exposure)? (Q6) | Were the outcomes measured in a valid and reliable way? (Q7) | Was the follow-up time reported and sufficient to be long enough for outcomes to occur? (Q8) | Was follow-up complete, and if not, were the reasons to loss to follow up described and explored? (Q9) | Were strategies to address incomplete follow up utilized?(Q10) | Was appropriate statistical analysis used? (Q11) |  |  |
| Abeysena et al. (2010) | Yes | Yes | No | No | No | Yes | Yes | No | Yes | No | No | 45.5 | High |
| Bell et al. (2008) | Yes | Yes | No | Yes | Yes | No | Yes | Yes | Yes | Yes | Yes | 81.8 | Low |
| Bonzini et al. (2009) | Yes | Yes | No | Yes | Yes | Yes | Yes | Yes | Yes | Yes | Yes | 90.9 | Low |
| Both et al. (2010) | Yes | Yes | No | Yes | Yes | Yes | Yes | Yes | Yes | Yes | Yes | 90.9 | Low |
| Burdorf et al. (2011) | Yes | Yes | No | Yes | Yes | Yes | Yes | No | Yes | No | Yes | 72.7 | Low |
| Celikkalp et al. (2017) | Yes | Yes | Yes | Yes | No | No | Yes | Yes | No | No | Yes | 63.6 | Moderate |
| Jansen PW et al. (2010) | Yes | Yes | No | Yes | Yes | Yes | Yes | Yes | Yes | Yes | Yes | 90.9 | Low |
| Kader et al. (2021) | Yes | Yes | Yes | Yes | Yes | Yes | Yes | Yes | Yes | Yes | Yes | 100 | Low |
| Knudsen et al (2017) | No | Yes | No | Yes | Yes | Yes | Yes | Yes | Yes | Yes | Yes | 90.9 | Low |
| Lawson et al. (2009) | Yes | Yes | No | No | No | No | Yes | Yes | Yes | No | No | 45.5 | High |
| Lee et al. (2017) | Yes | No | Yes | No | Yes | Yes | Yes | Yes | Yes | Yes | Yes | 80 | Low |
| Magann et al. (2005) | Yes | Yes | Yes | Yes | No | Yes | Yes | Yes | No | Yes | Yes | 81.8 | Low |
| Mocevic et al. (2014) | Yes | Yes | Yes | Yes | Yes | Yes | Yes | Yes | Yes | Yes | Yes | 100.0 | Low |
| Niedhammer et al. (2009) | No | No | Yes | Yes | No | No | Yes | Yes | No | No | Yes | 45.0 | High |
| Pompeii et al. (2005) | Yes | Yes | No | Yes | Yes | No | Yes | Yes | Yes | Yes | Yes | 90.9 | Low |
| Runge et al. (2013) | Yes | Yes | No | Yes | Yes | Yes | Yes | Yes | Yes | No | Yes | 81.8 | Low |
| Shirangi et al. (2009) | Yes | Yes | No | Yes | Yes | Yes | No | Yes | Yes | No | Yes | 72.2 | Low |
| Skroder et al. (2021) | Yes | Yes | No | Yes | Yes | Yes | Yes | Yes | Yes | Yes | Yes | 90.9 | Low |
| Snijder et al. (2012) | Yes | Yes | Yes | Yes | Yes | Yes | Yes | Yes | Yes | Yes | Yes | 100 | Low |
| Specht et al (2019) | Yes | Yes | Yes | Yes | Yes | Yes | Yes | Yes | Yes | Yes | Yes | 100 | Low |
| Stinson et al. 2003 | Yes | Yes | No | No | No | No | No | Yes | Yes | Yes | No | 45.0 | High |
| Takeuchi et al. (2014) | Yes | Yes | No | Yes | Yes | Yes | Yes | Yes | Yes | Yes | Yes | 90.9 | Low |
| Vrijkotte et al. (2021) | Yes | Yes | Yes | Yes | Yes | Yes | Yes | Yes | Yes | Yes | Yes | 100.0 | Low |
| Zhu et al. (2004) | Yes | No | No | No | No | Yes | Yes | No | Yes | No | Yes | 45.0 | High |
