# Supplementary material for: Maternal Occupational Risk Factors and Preterm Birth: A Systematic Review and Meta-Analysis
Source: Public Health Rev. 2023 Oct 23;44:1606085. doi: 10.3389/phrs.2023.1606085 (PMC10625911; doi:10.3389/phrs.2023.1606085)
Supplement: Supplementary file 3 [file Table3.DOCX]

| **The overall evidence assessment between the most common physical occupational risks and preterm birth** | | | | | | | | | |
| --- | --- | --- | --- | --- | --- | --- | --- | --- | --- |
| The association between prolonged standing and preterm birth | | | | | | | | | |
| No of studies | Study design | Limitations | Inconsistency | Indirectness of evidence | Imprecision | Publication bias | Effect (Cofounding, Effect size, large magnitude of effect) | Certainty of evidence | Strength of message |
| (n=9) | Observational studies | Not series | Not concern | Serious concern | Serious concern | Not serious | No reason to rate up | Very low | “Not enough evidence from the scientific literature to guide policymakers, clinicians, and patients” |
| The association between lifting and preterm birth | | | | | | | | | |
| No of studies | Study design | Limitations | Inconsistency | Indirectness of evidence | Imprecision | Publication bias | Effect | Certainty of evidence | Strength of message |
| (n=11) | Observational studies | Not serious | Serious concern | Serious concern | Serious concern | Not serious | No reason to rate up | Very low | “Not enough evidence from the scientific literature to guide policymakers, clinicians, and patients” |
| The association between physical workload and preterm birth | | | | | | | | | |
| No of studies | Study design | Limitations | Inconsistency | Indirectness of evidence | Imprecision | Publication bias | Effect | Certainty of evidence | Strength of message |
| (n=8) | Observational studies | Not serious | Not serious | Serious concern | Not serious | Not serious | Rate up  (Effect size and plausible confounding) | Moderate | “Practice considerations” (moderate) |
| The association between working hours and preterm birth | | | | | | | | | |
| No of studies | Study design | Limitations | Inconsistency | Indirectness of evidence | Imprecision | Publication bias | Effect | Certainty of evidence | Strength of message |
| (n=12) | Observational studies | Not serious | Not serious | Serious concern | Not serious | Not serious | Rate up  (Effect size and plausible confounding) | Moderate | ““Practice considerations” (moderate)” |
| The association between shift work and preterm birth | | | | | | | | | |
| No of studies | Study design | Limitations | Inconsistency | Indirectness of evidence | Imprecision | Publication bias | Effect | Certainty of evidence | Strength of message |
| (n=8) | Observational studies | Not serious | Not serious | Serious concern | Not serious | Not serious | Rate up  (Effect size and plausible confounding) | Moderate | “Practice considerations” (moderate) |
| The association between whole-body vibration and preterm birth | | | | | | | | | |
| No of studies | Study design | Limitations | Inconsistency | Indirectness of evidence | Imprecision | Publication bias | Effect | Certainty of evidence | Strength of message |
| (n=3) | Observational studies | Not serious | Not serious | Serious concern | Not serious | Not serious | Effect size and plausible confounder | Moderate | “Practice considerations” (moderate) |
